# Supplementary material for: Unraveling the mechanistic links between blood pressure regulation and calcium-magnesium homeostasis: Insights into hypertension, hyperparathyroidism, and mineral disorders
Source: PLoS One. 2026 Feb 4;21(2):e0341800. doi: 10.1371/journal.pone.0341800 (PMC12872021; doi:10.1371/journal.pone.0341800)
Supplement: Attachment — Submitted filename: rebuttal.pdf [file pone.0341800.s001.pdf]

Additional Editor Comments:

1. This computational model is primarily based upon the data in male rats. The authors should acknowledge the limitations for extrapolating the results in male rats to humans.

Reply: Yes, there are significant interspecies differences between rat and human in terms of Ca^{2+}, Mg^{2+}, and blood pressure regulation, including intestinal Ca^{2+} and Mg^{2+} absorption, bone remodeling, and baseline hemodynamics. As such, the translation from rat to human is not straightforward. While qualitative feedback structure and directionality of hormonal effects may translate well, other more quantitative predictions such as time constants and gain values may not. These limitations are now acknowledged in the Discussion.

2. It is unclear what other factors than Ca, Mg and calciotropic hormones, such as SNS tone, etc., are incorporated in the calculation model.

Reply: The blood pressure regulation model extends previously published frameworks by Leete et al., which themselves build on foundational models developed by Hallow et al., Karaaslan et al., and Guyton et al. These models describe the coupled interactions among the cardiovascular system, renal function, renal sympathetic nervous activity, and the renin–angiotensin endocrine axis. Because these components have been described in detail elsewhere, we do not reproduce all model equations or provide a complete description of every subsystem. Instead, we focus on those components that are directly regulated by calcium, magnesium, and calcitropic hormones. Additional text and references have been included in the Methods section to clarify how other physiological factors are represented within the model.

3. Descriptions about native vitamin D, D3, 25OHD and 1,25(OH)2D should be corrected. In addition, physiological concentrations of 1,25(OH)2D do not enhance bone resorption.

Reply: The usage of vitamin D, D3, 25(OH)D and 1,25(OH)2D3 has been corrected.

Yes, physiological concentrations of 1,25(OH)2D3 do not independently stimulate bone resorption but instead act permissively to support PTH-mediated resorption. We have revised Eq. (20) to better reflect this relationship and added a clarifying sentence immediately below the equation.

4. PTH and PTHrP act via PTH1R on the vascular wall to enhance vasodilation. How these facts reconcile with the authors' assumption that high levels of PTH enhance vasoconstriction?

Reply: Yes, you are correct that although PTH can elicit modest Ca^{2+} entry signals in vascular smooth muscle cells, the net integrated signaling downstream of PTH1R favors smooth muscle relaxation rather than contraction. Accordingly, we revised the formulation

describing the effect of PTH on vascular resistance (Eq. 28) such that vascular resistance increases by up to 20% at very low PTH levels and decreases by up to 20% at high PTH levels. Despite this vasodilatory influence, vascular resistance remains elevated in hyperparathyroidism because the vasoconstrictive effect of AT1R-bound angiotensin II predominates.

5. $[Ca2+]_{p\text{-}eq}$ and $[Mg2+]_{p\text{-}eq}$ should be more clearly defined.

Reply: My mistake. The numerator and denominator of the $[Mg^{2p}]_p/[Mg^{2p}]_{p\text{-}eq}$ fraction were reversed. This has now been corrected and the formulae should make more sense.

Reviewer #1:
1) In Figure 2, MAP is determined by cardiac output and peripheral vascular resistance, which is fine. But cardiac output seems to be a function of venous return/circulating volume here. Various factors including SNS tone, PTH, Ca and others affect cardiac rhythm and contractile function of the heart. I wonder how the current computational model could incorporate the factor of cardiac function.

Reply: The blood pressure regulation model is an expansion of published models by Leete et al., which is in turn based on the models by Hallow et al., Karaaslan et al., and Guyton et al. These models describe the interactions among the cardiovascular system, the renal system, the renal sympathetic nervous system, and the endocrine (renin-angiotensin) system. Since these models have been published, we did not reproduce all the model equations or describe every component, but only those that are directly regulated by Ca, Mg, and calcitropic hormones. We have added text in the Methods section and included references to make clear that other factors are represented in the model.

2) Page 7: 1,25D does not stimulate bone resorption at least in near-physiological concentrations. What is the rationale for the assumption that both PTH and 1,25D affect bone resorption?

Reply: Yes, you are correct that physiological concentrations of 1,25(OH)2D3 does not enhance bone resorption; they merely “permit” PTH-driven resorption. We have corrected Eq. (20) to better represent this effect, and added a sentence below that equation to clarify.

3) Page 8: The authors describe that they modeled the effect of PTH on vascular resistance by assuming that at very low PTH vascular resistance would decrease by 20% and at high PTH vascular resistance would increase by 20%. However, PTH is a well-known vasodilator. I wonder how this assumption could be justified.

Reply: Yes, you are correct that although PTH can increase Ca^{2+} entry signals in vascular smooth muscle cells, the net integrated signaling downstream of PTH1R strongly favors relaxation, not contraction. We have modified the term that describes the effect of PTH on vascular resistance (Eq. 28), such that at very low PTH vascular resistance would increase

by 20% and at high PTH vascular resistance would decrease by 20%. Vascular resistance still increases in hyperparathyroidism, because the vasoconstrictive effect of AT1R-bound Ang II dominates.

Reviewer #2:
Major
1. Because this model is based primarily on a male rat, it is possible that all the results cannot be applied to human. For example, calcium deficiency does not cause 30% decrease in serum calcium in humans. Still, the authors indicate that the model provides interactions that are consistent with diverse clinical observations. It should be mentioned which results can be specific for rats.

Reply: The reviewer is correct that there are significant interspecies differences between rat and human in terms of Ca^{2+}, Mg^{2+}, and blood pressure regulation, including intestinal Ca^{2+} and Mg^{2+} absorption, bone remodeling, and baseline hemodynamics. As such, the translation from rat to human is not straightforward. While qualitative feedback structure and directionality of hormonal effects may translate well, other more quantitative predictions such as time constants and gain values may not. These limitations are now acknowledged in the Discussion.

2. While PTH is shown to increase arterial resistance, several reports indicate that PTH is a vasodilator. How do you explain this?

Reply: Yes, you are correct that although PTH can increase Ca^{2+} entry signals in vascular smooth muscle cells, the net integrated signaling downstream of PTH1R strongly favors relaxation, not contraction. We have modified the term that describes the effect of PTH on vascular resistance (Eq. 28), such that at very low PTH vascular resistance would increase by 20% and at high PTH vascular resistance would decrease by 20%. Vascular resistance still increases in hyperparathyroidism, because the vasoconstrictive effect of AT1R-bound Ang II dominates.

3. Calcium and magnesium are shown to have opposite effects on RSBA and vascular resistance (p. 6, 8). It is not clear how effects of these ions can be calculated in the similar formulas (26 and 27). Maybe, [Ca2+]p-eq and [Mg2+]p-eq need to be more clearly explained.

Reply: My mistake. The numerator and denominator of the $[Mg^{2p}]_p/[Mg^{2p}]_{p-eq}$ fraction were reversed. This has now been corrected and the formulae should make more sense.

Minor
1. Vitamin D and vitamin D3 are not the same. 25(OH)D is not an inactive form of vitamin D3, but 25-hydroxylated product of vitamin D. Wordings concerning vitamin D should be confirmed.
Reply. The usage of vitamin D, D3, 25(OH)D and 1,25(OH)2D3 has been corrected.

2. Henle’s loop is a component of distal tubule (Figure 1).
Reply: In Fig. 2(A), the “proximal tubule” includes both the proximal tubule and the loop of Henle, to better correspond to Eq. 22. For clarify this unconventional representation is now noted in the caption.

3. It is not clear what right arterial pressure indicates (Figure 2).
Reply: That was a typo, our apologies. It should be right atrial pressure. The figure has been corrected.

4. References should be presented in a fixed format.
Reply: We have made the bibliography format consistent.
